# National Antimicrobial Consumption in Latin America and the Caribbean: Measurements and Results from 2019–2022

**DOI:** 10.3390/antibiotics14030240

**Published:** 2025-02-27

**Authors:** Gustavo H. Marin, Lucía Giangreco, Paola Lichtenberger, Cristian Dorati, Perla Mordujovich, Robin Rojas-Cortés, Tatiana Orjuela-Rodríguez, Pilar Ramón-Pardo, José Luis Castro, Danini Marin, Ana Ramirez, André Lacerda Ulysses de Carvalho, Silvia Boni, Julie Williams, Maria Francisca Aldunate-González, Mónica López-Peña, Shing Mi Ching Fung, Hugo Marín-Piva, Ismary Alfonso-Orta, Sunil Singh, Alex Rodríguez-Mejía, Alicia María Molina, Carmen Buzarquis, Hilda Mantilla-Ponte, Vanessa Matthew, Gracious M. James, Rajeev P. Nagassar

**Affiliations:** 1 University Centre of Pharmacology (CUFAR), UNLP, WHO-PAHO Col, La Plata 1900, Argentina; 2 Miller School of Medicine, University of Miami, Miami, FL 33136, USA; 3 Pan American Health Organization (PAHO), Washington, DC 20037, USA; 4 Independent Consultant, Washington, DC 20037, USA; 5 National Administration of Drugs, Food, and Medical Technology (ANMAT), Buenos Aires 1480, Argentina; 6 Barbados Drug Service, St. Michael 11464, Barbados; 7 Public Health Institute, Ministry of Health, Santiago 1000, Chile; 8 Ministry of Health and Social Protection, Bogotá 110311, Colombia; 9 Costa Rican Social Security (CCSS), San José 10105-1000, Costa Rica; 10 Center for State Control of Medicines, Equipment, and Medical Devices (CECMED), La Habana 11300, Cuba; 11 Ministry of Health, Region 2 002, Guyana; 12 Secretary of Health, Tegucigalpa 11101, Honduras; 13 Health Regulation Agency (ARSA), Tegucigalpa 11101, Honduras; 14 Ministry of Public Health and Social Welfare (MSPBS), Asunción 2160, Paraguay; 15 General Directorate of Medicines, Supplies, and Drugs (DIGEMID), Lima 15087, Peru; 16 Joseph Nathaniel France General Hospital, Basseterre KN0101, Saint Kitts and Nevis; 17 Alexandra Hospital, Charlestown KN0802, Saint Kitts and Nevis; 18 The Sangre Grande Hospital, The Eastern Regional Health Authority, Sangre Grande, Trinidad and Tobago; rpnagassar@gmail.com

**Keywords:** antimicrobial, consumption, resistance, Latin America, Caribbean, AWaRe

## Abstract

Antimicrobial resistance (AMR) represents a major threat to health with significant global economic and safety implications. One of the major drives of this resistance is the misuse and overuse of antimicrobials. **Background/Objectives:** In this sense, WHO proposed to it members to estimate the antimicrobial consumption (AMC) at each country level, in order to help local authorities in their decision making related to AMR control. Although this program is already installed worldwide, in the American continent, the rate of countries’ inclusion has been delayed. This paper describes the efforts of Latin American & Caribbean countries in terms of AMC local assessment. **Methods**: AMC data collection was done as per the GLASS tool proposed by WHO. Analysis was performed using Daily Defined Dose each one thousand inhabitants day (DID) for global, therapeutical group or each antibiotic. Access, Watch and target Reserve (AWaRe) WHO classification was applied after data collection. **Results**: 13 countries were included during the period 2019–2022. The global DID ranged from 2.55 DID to 36.26 DID. The trend of this consumption did downward along the years. One of the factors than impacted the AMC was the COVID-19 pandemic. The most problematic antimicrobial misuse was seen in certain beta lactams and macrolides, like ceftriaxone and azithromycin. Regarding the AWaRe classification, 5 out of 13 countries accomplished the target until 2023 of 60% consumption for “Access antimicrobial’s group by 2023). This data helped local health managers to propose changes for better control of the AMR problem (ceftriaxone usage limitations in Peru, antimicrobial law in Argentina, etc.). **Conclusions**: The first steps towards AMC country’s assessment has been initiated. The present work provided essential inputs to local health authorities for decision making related to AMR control. It will be necessary to keep applying this results in regulate antibiotic usage at country level, as well as enrolling more countries in the AMC project.

## 1. Introduction

Antimicrobial consumption in human health is the major driver for antimicrobial resistance (AMR) representing a major threat to global health policies with additional significant economic and social implications [1,2,3,4,5,6,7]. This is one of the main reasons that the World Health Organization (WHO), by the WHO Expert Committee on Selection and Use of Essential Medicines, created in 2017 the AWaRe antibiotic’s classification focusing in antimicrobial risk to develop resistance [8], and also released during 2024, its political declaration at the 79th United Nations General Assembly (UNGA) to ensure, by 2030, that the use of WHO Access group antibiotics is expanded from the 2023 global target, and in that regard, taking into account national contexts, aim to achieve at least 70 per cent overall human antibiotic use globally, through investing in and strengthening stewardship programs [9].

In order to identify areas in which actions or interventions are needed, data from surveillance of AMR and antimicrobial consumption (AMC) are essential. These data should be standardized, easy compared and exchanged to be used locally, nationally, and globally in policies development.

Since 2015, the Global Antimicrobial Resistance and Use Surveillance System (GLASS) launched by the World Health Organization (WHO) aims to ensure continuity of successful treatment and prevention of infectious diseases with effective and safe medicines that are quality-assured, used in a responsible way, and accessible to all who need them. For the first time, an integrated system had the capacity to collect and analyze both the national AMR surveillance data originated by the microbiology laboratories and the national AMC reported by the countries [10].

The specific component dedicated at monitoring AMC, referred here as GLASS-AMC, provides a common technical basis for setting up national surveillance systems on AMC that can produce reliable and comparable data at national and global levels [10]. The methodology is available to be used by all countries in order to become a standard national AMC surveillance system that provides the possibility to compare results among any nation.

However, even though the relation between AMR and AMC is well documented [11], and the WHO-AMC methodology is widely available, there is limited reporting on antimicrobial use in low-and-middle income countries (LMICs). In 2018, GLASS-AMC presented data results from 2015–2016 on the consumption of systemic antibiotics from 194 countries and areas, contributing to the understanding of how antibiotics are used there [12]. In the document, 31 of the countries were considered as low-income, and 106 as middle incomed. In the American region, only 6 out of the 35 countries of the Americas most of them are low or middle-low-income nations) were able to collect and share their data representing only 17% of the nations studied, compared to 85% representation from Europe. Those results prompted to question the reasons behind the low participation of countries in the Americas and raised the concern for possible limitations to implement the WHO methodology in LMIC.

WHO had committed to work closely with member states, donors, and development agencies to strengthen the global surveillance of AMC by providing technical support to LMIC without existing national surveillance systems.

Additionally, the Pan American Health Organization (PAHO), with its collaborating Center (CC) *University Center of Pharmacology* (CUFAR as per its Spanish acronym) from Argentina, supported countries in the Americas and the Caribbean to register the national consumption of antimicrobials, with the idea to train local professionals in data collection and install a common methodology for conducting the GLASS-AMC in the Region.

As part of this work, in 2022, the initial results were published, including 2019 data from Argentina, Chile, Colombia, Costa Rica, Paraguay and Peru [13]. The present paper, reports the progress of implementing the WHO methodology in 13 Latin American and Caribbean countries presenting data from 2019 to 2022.

## 2. Materials and Methods

This is a descriptive study of national AMC in Latin American and Caribbean countries. Full details are available in the WHO Methodology for the Surveillance of National Antimicrobial Consumption [14,15].

*Antimicrobials studied*: Antimicrobials included in the study corresponded to groups: J01—antibacterial for systemic use, A07AA—antibiotics for alimentary tract, and P01AB—nitroimidazole derivatives for protozoal diseases of the WHO Anatomical Therapeutic Chemical (ATC) classification system.

*Data collection tool, verification and validation*: AMC data was collected using a tool developed by the WHO based on Microsoft^®^ Excel [15], which has been implemented in several countries around the world since 2017. The instrument is updated annually taking into account modifications or new additions to the ATC/DDD system.

The template incorporates macro functions that facilitate the automated calculation of consumption for each antimicrobial individually, according to the route of administration. The quality control of the collected information, which included the identification of missing data (units, strength, codes) and/or errors, as well as the resolution of bugs, was manually carried out by an external audit performed by CUFAR. Uncompleted files were returned to the focal point of each country in order to fix the problem. Finally, the data collected was validated by each country in collaboration with CUFAR and PAHO.

*Data analysis*: Data was evaluated using the Anatomical Therapeutic Chemical as the classification system (ATC) and the Defined Daily Dose as a unit of measure (DDD), system developed by the WHO Collaborating Centre for Drug Statistics Methodology—Norwegian Institute of Public Health [16,17].

AMC was calculated for each antimicrobial drug dividing the total amount (in grams) consumed by the DDD value, as per the following formula:

No. of DDD = Total grams/DDD value

where, the total amount of each antimicrobial (total grams) is determined by adding the grams of active ingredient of the various formulations (for example, different concentrations of tablets, capsules and syrup formulations) and package sizes consumed, and the DDD value corresponds to that assigned to each medication by the WHO CC.

For comparative purposes, these data were adjusted according to the size of the population under study. The accepted standard metric for national AMC estimates is the DDD per 1000 inhabitants per day (DID).

DID = No. of DDD × 1000/population × 365

Total AMC was determined by country by year. Additionally, consumption was analyzed by therapeutic subgroup—according to the ATC classification -, as well as categorized by the WHO Access, Watch, Reserve (AWaRe) group [8], that classify the antibiotics into those three groups based on their clinical importance and the risk of their use promotes resistance. According to UNGA 2024 Declaration it is expected that countries can achieve that 70% of their antimicrobial consumption could be from Access group by 2030 [9].

*Training*: The coordinators and working groups from authorities of participating countries received training and instruction though the “Online Training on WHO Methodology for Antimicrobial Consumption Surveillance”, available on PAHO’s Virtual Campus for Public Health (VCPH) [18], as well as through virtual workshops and individual exchange meetings conducted by CUFAR.

*Period of the study*: The data was collected and analyzed annually each year calendar for the period between 1 January 2019 and 1 January 2023.

*Variables considered in the analysis*: Country, source of information, active ingredient according to the ATC classification system from groups J01, A07AA, and P01AB, number of packages, dosage form, concentration of the antimicrobial in the pharmaceutical presentation, AWaRe group, DDD (Defined Daily Doses), DID (DDD/1000 inhabitants/day), sectors (public, private, social security) and levels of the health system (ambulatory, hospital, total), and population under study.

*Unit of analysis*: Each country enrolled in the study was considered one unit of analysis.

*Sources of information*: Information used to analyze AMC data in each country was obtained from sources recommended by from local authorities (Table 1). These sources include data from pharmaceutical companies regarding production and imports, distribution or procurement records from Ministries of Health, and sales data from private pharmacies. Each country provides its data through its national authorities (Ministry of Health, the National Regulatory Authority, or the Pharmacovigilance Departments).

Furthermore, AMC data was obtained from two different sectors: public (which includes antimicrobials purchased by the government), and private (which includes antimicrobials purchased by third parties). The data collection depends on the availability of data for the counterpart sector. The procedure may involve separate collection (public and private) or aggregate collection (global).

Similarly, the data collected was stratified by the level of care provided by the institutions that procure the antimicrobials: hospital (antimicrobials purchased and dispensed by hospital pharmacies to hospitalized patients) or community (antimicrobials bought and dispensed by community pharmacies). Countries were able to disaggregate the data in community or hospital level or maintain it as a whole if differentiation is not possible (total level).

*Argentina*

National production and imports of antimicrobials are mandatorily reported by pharmaceutical companies to the National Administration of Drugs, Food and Medical Technology (ANMAT). These data were used as a source of information, representing the global AMC at the country level, including information from the public and private sectors, as well as hospital and community levels, without disaggregation.

*Barbados*

Customs records provided data on antimicrobial imports at the national level. Since Barbados does not have a local pharmaceutical industry, all antimicrobials used were imported, resulting in data corresponding to global AMC without disaggregation.

*Brazil*

Pharmacy sales records obtained from IQVIA (Formerly IMS Health) were used to reflect consumption in the private and community sector.

*Chile*

The observatory of the Central Supply of the National Health Services System (CENABAST), under the Ministry of Health, was used as the source of information. The data were representative of the public sector, without disaggregation between community and hospital consumption.

*Colombia*

Two different sources of information were used, which, when combined, allowed to obtained all the necessary data to calculate the AMC: the Marketing Authorizations from the National Institute of Surveillance of Medicines and Food (INVIMA) and the Drug Price Information System (SISMED), through which laboratories must report to the Ministry of Health all drugs sold and their prices, whether domestically manufactured or imported. The data obtained corresponded to the global AMC (public and private sectors, and hospital and community levels, without disaggregation).

*Costa Rica*

The information source used was the Accounting and Supplies Computer System (SICS) of the Costa Rican Social Security Fund (CCSS). Therefore, the data corresponds to the public sector, without disaggregation between community and hospital consumption.

*Cuba*

The database of the sole state-owned pharmaceutical distributor was used, including both domestic production and imports, thus corresponding to the public sector (community and hospital, without disaggregation).

*Guyana*

Procurement records from the Ministry of Health (Region 2) were used, corresponding to the public sector (community and hospital, without disaggregation).

*Honduras*

Distribution records from the Secretary of Health (SESAL), represented data from the public sector without disaggregation by level.

*Paraguay*

Procurement records from the Ministry of Public Health and Social Welfare (MSBPS) were used, corresponding to data from the public sector without disaggregation.

For 2019, different source of information was used (hospital records from selected public and private institutions, chosen based on convenience).

*Perú*

Two different sources of information were used, analyzing the data in an aggregated manner. They correspond to dispensing records from the Ministry of Health (MINSA)—obtained through the Medication Supply System (SISMED), under the General Directorate of Medicines, Supplies, and Drugs (DIGEMID)—and from the Social Security (EsSalud). The data are representative of the public sector, without differentiation between community and hospital consumption.

*Saint Kitts and Nevis*

The procurement records from the Ministry of Health were used, representing data from the public sector. While Saint Kitts’ data combined community and hospital consumption, Nevis’s data were analyzed separately for these two levels.

*Trinidad and Tobago*

Procurement records from the Eastern Regional Health Authority (ERHA) of the Ministry of Health are used, representing the public sector. The data was analyzed both aggregated and by levels (community or hospital) [19].

*Population under study*: Given that consumption was adjusted per population for comparative purposes, the population under study was defined for each country based on the information source utilized.

For Argentina, Barbados, and Colombia, where the data represented global AMC (both public and private sectors, community and hospital levels), the entire national population was considered for calculations.

In the case of Brazil, it is also assumed that the population under study is the entire country, as potentially all individuals can procure medicines from private pharmacies.

Cuba also evaluated consumption across the entire population, given the uniqueness of its completely public healthcare system. Hence, the percentage of population coverage used was 100%.

Chile, Costa Rica, Honduras, Paraguay, Peru, and Saint Kitts and Nevis, provided information sources from social security, thus representing the public sector. The percentages of public sector coverage, and therefore the denominator used for calculations, vary depending on the extent of the public sector within the healthcare system.

In the case of Guyana and Trinidad and Tobago, consumption of the public sector was assessed at a subnational level (Region 2 in Guyana; Eastern Region in Trinidad and Tobago).

National public and official population figures or projections from the statistical institutes of each country were used, as well as population coverage data provided by the corresponding Ministries of Health or social security agencies. In the case of Colombia, previous studies have used the national population provided by the National Administrative Department of Statistics (DANE per its Spanish acronym). However, for this analysis, the population data was sourced from the United Nations World Population Prospects (UN-WPP) 2022. In the case of Honduras, the UN-WPP population data was also used as a source.

*Ethical considerations*: No nominal patient data was collected. All the extracted data corresponded to primary sources of each institution on the amounts of antimicrobials consumed during the study period. The study protocol was validated by the Pan American Health Organization Ethics Review Committee (PAHOERC). Ref. No: PAHOERC.0317.01

## 3. Results

**Figure 1:**
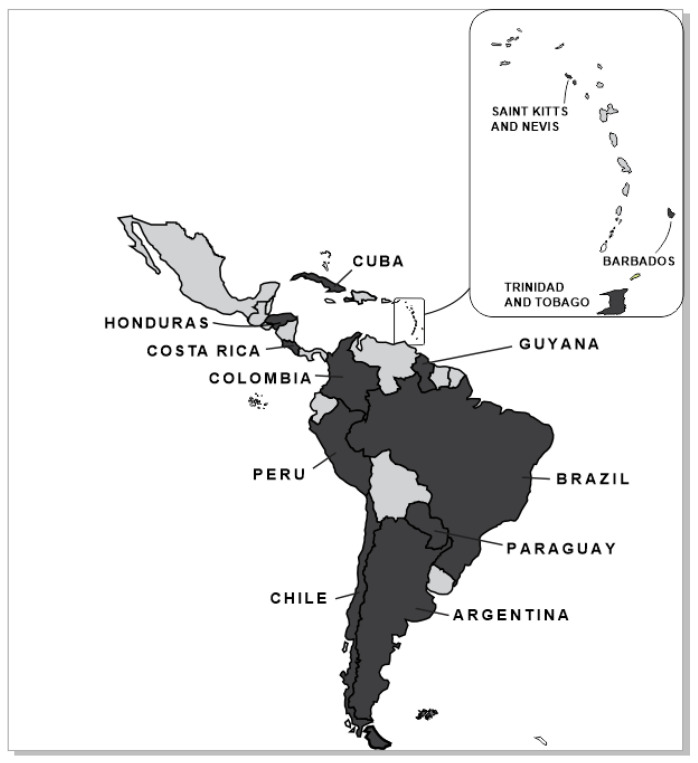
Participating Countries.

Participating countries were Argentina, Barbados, Brazil, Chile, Colombia, Costa Rica, Cuba, Guyana, Honduras, Paraguay, Peru, Saint Kitts and Nevis and Trinidad and Tobago (Figure 1).

### 3.1. Total AMC

Data analysis revealed a wide variability in total AMC, as shown in Table 2. Results ranged from 2.55 DID in Saint Kitts in 2022 to 36.26 DID in Argentina in 2019.

In all countries that monitored AMC during the four-year period of study, a tendency to decrease in consumption was observed: Argentina: −11.52 DID, (−31.8%); Chile: −1.07 DID, (−16.7%); Colombia: −1.36 DID, (−6.8%); Costa Rica: −4.26, (−33.4%); Guyana: −0.66 DID, (−5.0%); Honduras: −5.93 DID, (−45.4%); Peru: −2.17 DID, (−17.4%).

In Argentina, Chile, and Peru, the lowest consumption figures were observed during the COVID-19 pandemic years (2020 and/or 2021), with some increase in 2022 (however, not reaching the 2019 values). In contrast, Colombia and Guyana experienced an increase in AMC during the pandemic: Colombia: 6.45 DID, (+32.4%) including 2021; Guyana: 12.89 DID, (+98.2%) including 2020, but in 2022, consumption decreased, reaching the lowest figures of the period.

In Costa Rica and Honduras, AMC decreased during the 2020–2021 period, and continue to decline along the years, with even lower figures than during the pandemic.

Barbados monitored AMC in 2020 and 2022. Consumption increased during this period (2.8 DID, +18.2%).

The AMC in Trinidad and Tobago from 2019 to 2021 showed an increase in 2020 (0.61 DID, +9.9%), followed by a decrease during 2021. No data for 2022 was available.

In Cuba, the years 2021 and 2022 were monitored. During that period, consumption decreased from 13.96 to 11.74 DID (−15.9%).

In Paraguay, there was an increase in total AMC (3.29 DID, +38.9%) during the period 2020–2022. Although data was available for 2019, since the source was different, that year was not used for the analysis.

### 3.2. AMC by Therapeutic Subgroups

The analysis of AMC according to therapeutic subgroups, following the ATC classification, is detailed in Table 3.

In most countries, penicillin and their derivatives (J01C) were the most consumed group, with the exception of Brazil during 2020, where macrolides (J01F) were the majority. In Cuba, macrolides were the most consumed group during 2021 and 2022. During 2022, in the island of Saint Kitts, cephalosporins (J01D, other beta-lactams) were the most consumed group, while in Barbados, the group of sulfonamides and trimethoprim (J01E) was on top.

In Argentina, penicillin and their derivatives were the most consumed group throughout the evaluated period (46.5–51.6% depending on the year). During 2019, the second most used group was nitroimidazole derivatives (P01AB), accounting for 13.6%, followed by macrolides and lincosamides (13.3%). In 2020 and 2021, macrolides ranked second (20.3% and 21.1%, respectively), and quinolones (J01M) third (10.9% and 11.3%). In 2022, penicillins and macrolides remained in first and second place, while the group of other beta-lactams (cephalosporins, monobactams, and carbapenems) displaced quinolones to third place (7.9%).

In Barbados in 2020, the most consumed group of antimicrobials was penicillin and their derivatives (34.0%), followed by tetracyclines (J01A) (26.3%), and in third place, macrolides (16.8%). In 2022, the most commonly used antimicrobials were sulfonamides and trimethoprim in first place (30.4%), followed by penicillins (29.5%), and then tetracyclines (16.2%).

In Brazil, during 2019, penicillins and their derivatives were the most consumed group of antimicrobials (38.2%), followed by macrolides (19.8%). In 2020, this trend reversed, with macrolides taking first place with 31.7%, while penicillins and their derivatives accounted for 30.4%. Quinolones maintained third place in both periods, decreasing from 19.5% in 2019 to 16.8% in 2020.

In Chile, penicillin and their derivatives were the most consumed group throughout the 2019–2022 period, with a range of between 44.8% and 36.7% depending on the year. Macrolides consistently appeared in second place (17.5–23.4%). In 2019, quinolones ranked third (9.1%), while the other beta-lactams group ranked third in 2020 and 2022 (10.9% and 11.5% respectively). In 2021, the “other antibiotics” group (J01X) ranked third.

The analysis of Colombia revealed that penicillin and their derivatives were the most frequently consumed group throughout the entire period studied (30.5–39.3%), followed by macrolides (16.2–22.6%). Quinolones ranked third in 2019 and 2022 (10.6 and 11.5%, respectively), while the other beta-lactam group ranked third in 2020 and 2021 (13.4 and 10.4%).

In the case of Costa Rica, penicillin and their derivatives occupied the first place during the four years monitored (19.8–26.0%), followed by the group of other beta-lactams (16.6–18.1%). During 2019 and 2022, tetracyclines and the group of sulfonamides and trimethoprim (J01E) ranked third, with minimal difference between them, while in 2020 and 2021, tetracyclines predominated over sulfas.

The analysis of data from Cuba revealed that macrolides constituted the most consumed antimicrobials during the 2021–2022 period, with a range of 29.0–37.1%. This was followed by the group of other beta-lactams (20.8–21.4%). Third place was taken by penicillin and their derivatives during 2021 (19.9%), and quinolones in 2022 (15.6%).

In Guyana, penicillin and their derivatives were the most consumed group during the four years evaluated (34.8–48.5%), followed by macrolides (15.2–28.1%). In third place, during 2019, the most consumed group was sulfonamides and trimethoprim (13.9%), in 2020 it was other beta-lactams (11.4%), and in 2021 and 2022, quinolones (17.6% and 17.6%, respectively).

In Honduras, penicillin and their derivatives were also the most consumed anti-microbials during the study period (39.4–47.8%). In 2019, the second place was occupied by sulfonamides and trimethoprim (18.4%), followed by macrolides and lincosamides (13.2%). In the 2020–2021 period, macrolides moved up to second place, reaching 19.3% in 2020 and 26.3% in 2021, while sulfonamides and trimethoprim ranked third (15.0% in both years). Finally, in 2022, the pattern of antimicrobial consumption was again similar to the one in 2019.

In Paraguay, it was observed again that penicillins occupied the first place during the evaluated period (37.7–45.4%). Then, macrolides ranked second (23.2–26.3%), and the group of other beta-lactams third (14.3–19.0%).

In the case of Peru, the data also showed that penicillin and their derivatives were the most consumed group in all monitored years (29.9–35.9%), followed by macrolides (16.0–23.3%). The third place was occupied by quinolones in 2019, 2020, and 2022 (12.4–14.0%), but in 2021, the consumption of other beta-lactams predominated (13.6%).

In Saint Kitts and Nevis, each island conducted its own analysis, showing somewhat different consumption profile results. In the case of Saint Kitts, the most consumed group was other beta-lactams (23.5%), followed by quinolones (21.5%), and penicillin and their derivatives (18.9%) in third place. On the other hand, Nevis data showed that penicillins were the most consumed group (26.9%), followed by other beta-lactams (18.2%), and in third place, macrolides (17.5%).

In Trinidad and Tobago, throughout the study period, penicillins were the most consumed antimicrobials (38.4–45.9%), followed by the group of other beta-lactams (20.9–31.9%). In third place, in 2019 and 2021, quinolones ranked (20.5% and 11.3%, respectively), while in 2020, the group of sulfonamides and trimethoprim (18.5%) prevailed [19].

### 3.3. AMC According to AWaRe Classification

The analysis of AMC according to the AWaRe classification reveals that in only five out of the thirteen included countries, the consumption of the Access group reached the 60% recommended by the WHO/UNGA throughout the study period (even if since 2024, according to WHO recommendation this goal will be 70%): Argentina (63.2–78.1%), Barbados (72.6–84.0%), Colombia (65.7–74.4%), Costa Rica (83.1–86.5%) and Honduras (63.9–77.2%).

Chile reached the 60% target for the Access group between 2019 and 2021 (62.0–64.3%), but in 2022 the consumption of this group decreased to 59.8%.

In the case of Guyana, during 2021, the consumption of this group decreased to 54.7%, although during the rest of the years it always remained above the 60% target (65.6–72.3%). A similar situation was observed in Peru: in 2020, the consumption of the Access group decreased to 58.3%, but in other years of the period, the recommended indicator was met (61.0–66.6%).

In Brazil, the consumption of the Access group decreased from 58.3% in 2019 to 49.8% in 2020. In the same year, the Watch group consumption reached 50.2%, overtaking that of the Access group.

In Cuba during 2021, antimicrobials in the Access group accounted for 52.4% of total consumption, while the Watch group accounted for 47.3%. In 2022, the latter group increased to 59.3% of the total, while the Access category accounted for 40.7%.

In Saint Kitts, the consumption of the Watch group (51.1%) also exceeded that of the Access group (48.2%), while in Nevis, although the consumption of antimicrobials in the Access group did not reach the 60% target (58.9%), it was higher than the consumption of the Watch group (41.1%).

In Trinidad and Tobago, during 2020, the consumption of the Access group was 64.6% (and the consumption of the Watch group 35.3%), but in 2019 and 2021, the 60% consumption of Access antimicrobials was not reached, with the consumption of the Watch group being predominant (56.5% and 50.3%, respectively).

In Paraguay, the consumption of the Access group varied between 29.2% and 30.4% depending on the year, while the consumption of the Watch group ranged from 41.0% to 46.5%. The significant consumption of antimicrobials not included in the AWaRe categories, fluctuating between 24.2% and 28.5%, is due to the fact that all consumption of amoxicillin with beta-lactamase inhibitors (J01CR02) corresponds to the amoxicillin/sulbactam combination, a combination not included in the Access group.

The consumption of the Reserve group remained consistently below 0.2% in all countries throughout the study period, with the exception of Barbados in 2022, where this group accounted for 0.4% of the total consumption, and Chile also in 2022 (0.3%).

**Figure 2:**
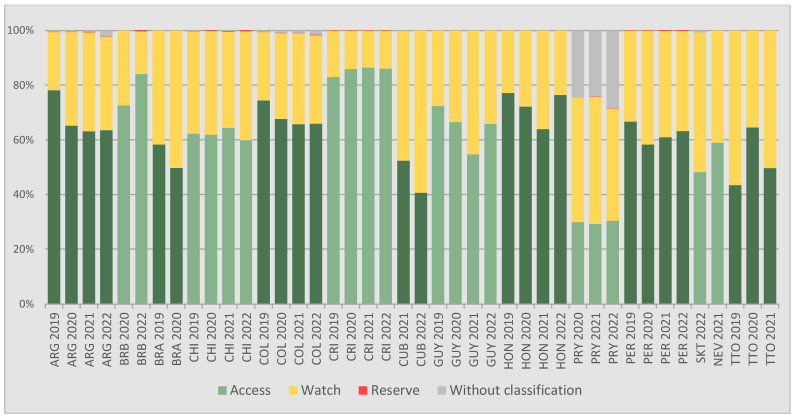
AMC according to WHO AWaRe classification, for each country and year, expressed as a percentage (%) of the total consumption (based on total AMC measured in DID (DDD/1000 inhab./day). The red line indicates the target proposed by the WHO: at least 60% of total consumption should correspond to antimicrobials from the Access group.

Table 4 and Figure 2 present the analysis of AMC according to the AWaRe classification.

## 4. Discussion

Measuring and monitoring AMC provides valuable information to understand the patterns and trends of antimicrobial use which is essential for designing effective strategies to containt AMR [14].

This paper provides information about AMC from the American continent, where previously only 17% of the countries in this region shared their data [12].

Following the pilot phase and the first WHO-AMC 2018 report [12], WHO published the “GLASS methodology for surveillance of national antimicrobial consumption” [14,15], hereafter referred to as the “GLASS-AMC methodology”, as main surveillance framework [1].

This methodology that can be integrated in the package of tools to assist the national strategy on optimizing antimicrobial use (AMU) (for example, national action plans on AMR), elements that can provide information on quantities and types of antimicrobials consumed in order to guide the prescribers and decisions of policy-makers to optimize the access and rational use of antimicrobials [20]. Furthermore, such measurements aid in resource allocation by guiding investments in healthcare infrastructure and antimicrobial stewardship initiatives. At the regional and global level, the GLASS-AMC provides a methodology common to all countries for the collection, analysis, and reporting of national AMC data, that is reliable and comparable with national consumption data over time and between countries as well, with animal and agricultural consumption data.

The last GLASS report from 2022 [21], includes information on the progress of the “GLASS methodology” implementation and show results of 26 countries that submitted data for the year 2020 from the African, Eastern Mediterranean, Euro-pean, South-East Asia, Western Pacific and the American regions. Only two American countries were included in that report (Colombia and Peru). The median value of the overall consumption of antibacterial was 16.6 (range, 12.3–31.2) DID. A higher fluctuation among nations was observed in the African region (median, 15.3 [range, 3.6–58.2]), South-East Asia and West Pacific Regions (median, 15.3 [range, 9.5–57.4]). All countries in the Eastern Mediterranean Region reported a consumption >29 DID (median, 31.8 [range, 29.4–53.6]). The median value for the six European countries was 15.3 (range, 9.2–30).

The results provide a more detail picture of AMC in the Americas, including 13 countries and a period of 4 years. The median value for AMC was 11.0 DID for the period 2019–2022; while in 2019 that index was 12.8 DID (range 6.2–36.3), in 2020, 10.5 (5.7–26.0); in 2021, 9.2 (3.6–26.4), and in 2022, 11.7 (2.6–24.7). This level of consumption was slightly lower than the one observed in other regions (i.e., 2019; 10.84 in America; 19.6 DID in Europe). However, it exists a great variability among nations either in America (in 2019, values range varied from 6.62 up to 36.6 DID in Trinidad & Tobago and Argentina respectively) or in Europe (range observed was 10.6 to 33.2 DID in Switzerland and Turkey) data that reflex the heterogeneity of AMC among countries [20]. This variability might be explained by prescription culture, affordability to access to certain antibiotics, and local markets that push and imposes some antimicrobials.

The European Center for Disease Prevention and Control (ECDC) is another source for AMC annual surveillance data. They publish their annual results and trends on its website [21]. According to this data, the regional trend in Europe DDD/1000 inhabitants per day for the years 2018 to 2022 for the ECDC corresponds to 21.6, 21.0, 17.9, 18.1 to 21.5 respectively (group J01). Countries with the highest AMC corresponds to Cyprus, Greece and Romania followed by Bulgaria and France with the lowest AMC among the Netherlands, Sweden, Austria, Estonia, Slovenia and Finland. In our work, it is observed that the results measured in DDD/1000 inhabitants per day in Brazil, Chile, Costa Rica, Honduras, Paraguay, Peru, Saint Kitts, Nevis and Trinidad and Tobago are lower when compared to the countries with the lowest AMC in the European region (less than 10 DDD per 1000 inhabitants per day). Countries like Argentina and Colombia have AMC similar to those in France, Spain or Italy (21–26 DDD per 1000 inhabitants/day) and Barbados and Cuba AMC results, are compared to data exhibited by Finland, Hungary and Norway (12–16 DDD per 1000 inhabitant per day). Independently of the AMC results, the trend over years is very similar. There is a marked decrease in the AMC during the COVID-19 pandemic, with an increased trend from 2021 on, to levels similar to those seen in 2018–2019.

These trend and data obtained in our own work, was confirmed by a systematic review that compared the effect of the pandemic on overall and individual antibiotic consumption in 2020 with 2019 [22]. This study demonstrated that during the COVID-19 pandemic, all community- and national-level studies reported an overall decrease in antibiotic consumption. This situation could be explained because the compulsorily isolation imposed on the community, and the non-pharmaceutical prevention measures applied, reduced the interpersonal contact, decreasing the transmission of infectious disease, mainly respiratory infections. This report confirms that the trends on AMC in LMIC was similar to the ones in high income ones [23].

Concerning the WHO AWaRe categories, the index for Access antibacterial group, which should be >60% (70% since 2024) of the total consumption, was only accomplished by 5 out of the 13 countries. Interestingly, not necessarily the countries with low AMC had an increased percentage of antimicrobial use in the Access group. That is the case of Trinidad and Tobago where the AMC in 2019 was 6.19 DDD per 1000 inhabitants per day but with 56.5% of the antimicrobials used in the Watch category group, in contrast to Argentina in 2019, where the AMC was 36.6 DDD per 1000 inhabitants per day, with 78% of the antimicrobials prescribed in the Access group. Because the situation described, WHO-AWaRe goals become a challenge for the futures years. It is not easy to radically change the culture of prescription in each country. We believe that it could be accomplished since although several countries are actually under the optimal percentage in Access group, most of them are really close the goal to the new target of 70% proposed at the 79th United Nations General Assembly.

In relation to programs focused in AMR control training (like PROA-PAHO which is a program for optimizing antimicrobial use, based on stewardship actions at hospital level) and its correlation with AMC, it could be said that in those countries with strong adherence to PROA, such as Chile and Peru; consumption rate is low to moderate.

However, it is important to remark that the PROA is performed at the hospital level, that is why, countries with less population and little outpatient consumption might especially be beneficiated from programs that propose the rational use of antimicrobials (like PROA). This could be the situation in Caribbean countries where the AMC is concentrated in few hospitals and the community consumption is low, hence, a strong adherence to AMR control programs may have excellent results.

Focusing in certain antimicrobials subgroups with high consumption like cephalosporins, it could be mention that the most used drug in this group was ceftriaxone. Either in Argentina (DID 0.16) and Peru (0.35 DID), an increasing trend along the years (+8.4% and +13.6% respectively) was observed, while at the same time, it existed a reduction in consumption of another drug like cefazoline during the same period (Argentina, −3.1%; Peru, −20.5%) pattern that might be explain by the irrational use of ceftriaxone in surgery prophylaxis or an excessive ambulatory use. Furthermore, increases in ceftriaxone consumption were noted in other countries in 2020 and/or 2021 (Chile, 16.3%; Colombia, 119.3%; Guyana, 1634.2%; Honduras, 31.8%; Paraguay, 102.1%), although in these cases, a return to pre-pandemic values was registered in the year 2022. Again, in some of these nations, cefazoline consumption was reduced. According to the national focal points, there was a lack of supply of 1st generation cephalosporins, which is usually used in pre-surgical prophylaxis. Due to the absence of market offers of cephalothin and other cephalosporin from the same group, ceftriaxone was inappropriately used to replace the first-generation cephalosporin in surgical prophylaxis, increasing in 221.9% its overall consumption.

Due to this situation, some countries like in Peru, health authorities are taking measures in order to control the excess of ceftriaxone in certain hospitals where the highest consumption was detected.

In other counties like Argentina, a National Law that controls sales of antimicrobials was enacted in 2022.

A pending task will be to associate these levels of consumption detected in this study, with the local laboratory surveillance data on AMR. Although some countries have begun to generate this type of data [11,23], there is still a gap in this regard.

Nonetheless, there is a recent publication on the relationship between AMR and AMC using data from the GLASS report published for 2022 [23]. This paper showed that a reduction in AMC is strongly associated with a decrease in AMR rates specially in high income countries; even more, it exist differences among regions of the same country showing that where the overuse of certain antimicrobial correlates to a high AMR to that same drug. This statement may not always be case in certain LMIC countries where low AMC was not associated with lower AMR (i.e., Bhutan) suggesting that other factors might be involved. These factors could be Antimicrobial Stewardship programs through national action plans and strategies, enforcing regulatory policies for AMC, multisectoral and one-health collaborations, and AMR/AMC surveillance systems; all elements that may lead to the overall reductions of both AMC and AMR rates but that they may not be always available.

That is why, it is necessary to recall this type of data in each country. In this sense, the information obtained from our study becomes relevant, since in American region, most of countries are considered LMIC, reminding the concept of the importance of surveillance regularly reporting their individual data in order to used it, in prioritizing activities and targeted individual nation’s AMR concerns.

By monitoring AMC at the national level, policymakers can identify areas of overuse or misuse and implement targeted interventions to promote rational prescribing practices. Additionally, tracking AMC helps assess the impact of existing antimicrobial stewardship programs and identify areas for improvement. Furthermore, such measurements aid in resource allocation by guiding investments in healthcare infrastructure and antimicrobial stewardship initiatives.

## 5. Conclusions

Measurement of AMC is crucial for several reasons; it provides valuable data to understand the patterns and trends of antimicrobial use, which is essential for designing effective strategies to prevent AMR. The results obtained in this study presents data from the 13 countries in the Americas on their AMC in terms of DDD/1000 inhabitants per day (DID) over a 4 years period, showing great heterogeneity among them (2.55 DID to 36.26 DID). One of the biggest misuses of antimicrobials seen was the ceftriaxone, showing unexplained raise of consumption in several countries due to irrational prophylaxis or ambulatory infections that should be treated with other and simplest antibiotic options.

Regarding the AWaRe classification it was detected that only 5 out of the 13 countries accomplished the target (valid until year 2023) of 60% consumption for “Access antimicrobial’s group. Even in countries with very low AMC, the same situation was seen, suggesting an opportunity for Antimicrobial Stewardship Programs like PROA offered by PAHO.

The efforts made by the countries that participated in this project promote the commitment of others in order to put the Region of Americas at better levels of AMC surveillance, as in other regions of the world.

## Figures and Tables

**Table 1:** Sources of information, health system sector and level, and population under study for each country and year.

	Source of Information	Sector			Nivel			Covered Population				% Covered Population of Total Country Inhabitants			
		Global	Public	Private	Total	Hospital	Community	2019	2020	2021	2022	2019	2020	2021	2022
**Argentina**	Local manufacturers and imports (ANMAT)	X			X			44,938,712	45,376,763	45,808,747	46,234,830	100			
**Barbados**	Imports	X			X			NR	280,693	NR	281,635	NR	100	NR	100
**Brazil**	Pharmacy sales (IQVIA)			X			X	210,147,125	210,592,714	NR	NR	100	100	NR	NR
**Chile**	Procurement records (CENABAST)		X		X			14,903,628	14,982,898	15,233,814	15,613,584	78.0	77.0	77.4	81.7
**Colombia**	Local manufacturers and imports (SISMED—INVIMA)	X			X			50,187,406	50,930,662	51,516,562	51,874,024	100			
**Costa Rica**	Procurement records (CCSS)		X		X			4,608,402	4,692,270	4,693,42	4,805,698	91.1	91.8	90.9	92.2
**Cuba**	Distribution records (MINSAP—CECMED)	X			X			NR	NR	11,147,405	11,101,363	NR	NR	100	
**Guyana ^1^**	Procurement records		X		X			46,810				6.30			
**Honduras**	Distribution records (SESAL)		X		X			8,054,342	8,196,908	8,334,800	8,475,736	75	75	75	75
**Paraguay ^2^**	Procurement records (MSPBS)		X		X			5,082,767	5,403,240	5,448,601	5,418,836	71.9	74.5	74.1	72.7
**Perú**	Dispensing records(MINSA + EsSalud)		X		X			25,062,491	25,448,239	25,800,572	27,385,294	78			82
**Saint Kitts**	Procurement records		X		X			NR	NR	NR	25,630	NR	NR	NR	100
**Nevis**	Procurement records		X		X	X	X	NR	NR	12,272	NR	NR	NR	100	NR
**Trinidad & Tobago ^3^**	Procurement records		X		X	X	X	120,000			NR	8.6			-

NR: No reported data. ^1^ Guyana AMC data was limited to region 2. ^2^ Paraguay AMC data for the year 2019 corresponds only to the hospital sector. ^3^ Trinidad and Tobago AMC data was limited to the Eastern Regional Health Authority (ERHA). ANMAT: National Administration of Drugs, Food, and Medical Technology of Argentina; CENABAST: Central Supply of the National Health Services System of Chile; SISMED: Drug Price Information System of Colombia; INVIMA: National Institute of Surveillance of Medicines and Food of Colombia; CCSS: Costa Rican Social Security; MINSAP: Ministry of Public Health of Cuba; CECMED: Center for State Control of Medicines, Equipment, and Medical Devices of Cuba; IHSS: SESAL: Secretary of Health of Honduras; MSPBS: Ministry of Public Health and Social Welfare of Paraguay; MINSA: Ministry of Health of Perú; EsSalud: Social Health Insurance of Perú.

**Table 2:** Total AMC for each country and year, expressed in DID (DDD/1000 inhab./day), and average consumption for the period 2019–2022.

	2019	2020	2021	2022	Average Total Consumption
**Argentina**	36.26	17.42	17.12	24.74	23.88
**Barbados**	NR	15.40	NR	18.20	16.80 ^d^
**Brazil**	7.05	7.06	NR	NR	7.06 ^d^
**Chile**	6.41	5.67	4.12	5.34	5.39
**Colombia**	19.90	22.51	26.35	18.54	21.83
**Costa Rica**	12.76	8.89	9.17	8.50 ^e^	9.83 ^e^
**Cuba**	NR	NR	13.96	11.74	12.85 ^d^
**Guyana** ^a^	13.13	26.02	14.02	12.47	16.41
**Honduras**	13.05	12.18	9.12	7.12	10.37
**Paraguay**	3.38 ^f^	8.45	9.64	11.74	9.94 ^g^
**Perú**	12.50	8.08	8.81	10.33	9.93
**Saint Kitts**	NR	NR	NR	2.55	2.55 ^d^
**Nevis** ^b^	NR	NR	3.55	NR	3.55 ^d^
**Trinidad and Tobago** ^a,b,c^	6.19	6.80	4.61	NR	5.87 ^d^

NR: No reported data. ^a^ Guyana AMC data was limited to Region 2, and for Trinidad and Tobago to the Eastern Regional Health Authority (ERHA). ^b^ The data presented corresponds to total consumption (hospital + community). ^c^ Only monitored a specific group of antimicrobials, chosen for convenience. ^d^ The average total consumption includes data only from the years evaluated. ^e^ There is a potential underestimation of AMC for Costa Rica during the year 2022, and therefore in the average consumption over the entire period, as the informatic systems of the CCSS were impacted by a cyberattack during that year. ^f^ The 2019 AMC data was limited to hospital level. ^g^ The average total consumption indicated is for the period 2020–2022. Data for 2019 were excluded to obtain the average total consumption, as they are hospital data.

**Table 3:** AMC categorized according to ATC classification, for each country and year, expressed in DID (DDD/1000 inhab./day) and as a percentage (%) of the total consumption.

		Intestinal antiinfectives (A07A)	Tetracyclines (J01A)	Amphenicols (J01B)	Beta-lactams, penicillins (J01C)	Other beta-lactams (J01D)	Sulfonamides and trimethoprim (J01E)	Macrolides (J01F)	Aminoglycosides (J01G)	Quinolones (J01M)	Other antibacterials (J01X)	Agents against amoebiasis and other protozoal diseases (P01AB)	Total
**Argentina**	**2019**	0.25 (0.7)	1.59 (4.4)	0	18.72 (51.6)	2.14 (5.9)	0.66 (1.8)	4.84 (13.3)	0.06 (0.2)	2.51 (6.9)	0.57 (1.6)	4.93 (13.6)	36.26 (100)
	**2020**	0.12 (0.7)	0.55 (3.2)	0	8.20 (47.1)	1.67 (9.6)	0.69 (4.0)	3.53 (20.3)	0.07 (0.4)	1.90 (10.9)	0.47 (2.7)	0.2 (1.2)	17.42 (100)
	**2021**	0.14 (0.8)	0.65 (3.8)	0	7.95 (46.5)	1.47 (8.6)	0.63 (3.7)	3.61 (21.1)	0.06 (0.3)	1.93 (11.3)	0.47 (2.7)	0.5 (2.9)	17.12 (100)
	**2022**	0.15 (0.6)	0.69 (2.8)	0	12.24 (49.5)	1.95 (7.9)	0.67 (2.7)	6.26 (25.3)	0.07 (0.3)	1.79 (7.2)	0.62 (2.5)	0.3 (1.2)	24.74 (100)
**Barbados**	**2020**	0	4.05 (26.3)	0	5.23 (34.0)	2.59 (16.8)	0.46 (3.0)	1.78 (11.6)	0.08 (0.6)	0.58 (3.8)	0.09 (0.6)	0.54 (3.5)	15.40 (100)
	**2022**	0	2.95 (16.2)	0	5.37 (29.5)	1.03 (5.6)	5.53 (30.4)	0.97 (5.3)	0.02 (0.1)	0.83 (4.6)	0.16 (0.9)	1.35 (7.4)	18.20 (100)
**Brazil**	**2019**	0	0.47 (6.6)	<0.01	2.69 (38.2)	0.80 (11.3)	0.29 (4.2)	1.40 (19.8)	0.01 (0.1)	1.38 (19.5)	<0.01	0.01 (0.2)	7.05 (100)
	**2020**	<0.01	0.47 (6.6)	<0.01	2.14 (30.4)	0.72 (10.2)	0.29 (4.1)	2.24 (31.7)	<0.01	1.18 (16.8)	<0.01	0.01 (0.2)	7.06 (100)
**Chile**	**2019**	0.04 (0.6)	<0.01	0	2.87 (44.8)	0.53 (8.2)	0.20 (3.1)	1.50 (23.4)	0.03 (0.5)	0.58 (9.1)	0.49 (7.6)	0.17 (2.7)	6.41 (100)
	**2020**	0.05 (0.8)	<0.01	0	2.60 (45.8)	0.62 (10.9)	0.20 (3.6)	1.19 (20.9)	0.06 (1.0)	0.58 (10.2)	0.25 (4.4)	0.14 (2.5)	5.67 (100)
	**2021**	0.11 (2.6)	0.10 (2.5)	0	1.49 (36.1)	0.38 (9.3)	0.26 (6.2)	0.72 (17.5)	0.05 (1.2)	0.44 (10.6)	0.47 (11.5)	0.10 (2.5)	4.12 (100)
	**2022**	0.16 (3.1)	0.15 (2.8)	0	1.96 (36.7)	0.61 (11.5)	0.07 (1.3)	1.05 (19.7)	0.06 (1.1)	0.58 (10.8)	0.56 (10.5)	0.13 (2.4)	5.34 (100)
**Colombia**	**2019**	0.21 (1.1)	1.66 (8.3)	0	7.82 (39.3)	2.10 (10.6)	0.95 (4.8)	3.22 (16.2)	0.15 (0.8)	2.11 (10.6)	1.00 (5.0)	0.68 (3.4)	19.90 (100)
	**2020**	0.31 (1.4)	1.68 (7.5)	0	6.85 (30.5)	3.02 (13.4)	0.80 (3.5)	5.08 (22.6)	0.16 (0.7)	1.89 (8.4)	1.50 (6.6)	1.22 (5.4)	22.51 (100)
	**2021**	0.35 (1.3)	1.94 (7.4)	0.04 (0.1)	8.74 (33.2)	2.73 (10.4)	0.93 (3.5)	5.25 (19.9)	0.22 (0.8)	2.71 (10.3)	1.88 (7.1)	1.57 (5.9)	26.35 (100)
	**2022**	0.36 (1.9)	0.87 (4.7)	0.04 (0.2)	7.06 (38.1)	1.54 (8.3)	0.33 (1.8)	3.79 (20.5)	0.09 (0.5)	2.13 (11.5)	1.87 (10.1)	0.46 (2.5)	18.54 (100)
**Costa Rica**	**2019**	<0.01	1.88 (14.8)	0	3.31 (26.0)	2.12 (16.6)	1.88 (14.8)	1.67 (13.1)	0.10 (0.8)	0.29 (2.2)	1.18 (9.2)	0.32 (2.5)	12.76(100)
	**2020**	<0.01	1.57 (17.7)	0	1.88 (21.2)	1.60 (18.0)	1.46 (16.4)	0.82 (9.3)	0.07 (0.8)	0.22 (2.5)	1.03 (11.6)	0.22 (2.5)	8.89 (100)
	**2021**	<0.01	1.64 (17.9)	0	1.81 (19.8)	1.66 (18.1)	1.59 (17.4)	0.71 (7.8)	0.08 (0.8)	0.25 (2.7)	1.16 (12.7)	0.26 (2.8)	9.17 (100)
	**2022** ^d^	<0.01	1.42 (16.7)	0	1.87 (22.1)	1.48 (17.5)	1.41 (16.6)	0.84 (9.9)	0.07 (0.8)	0.17 (2.1)	1.00 (11.7)	0.23 (2.7)	8.50 (100)
**Cuba**	**2021**	0	0.26 (1.9)	<0.01	2.78 (19.9)	2.90 (20.8)	1.10 (7.9)	4.05 (29.0)	0.15 (1.1)	1.66 (11.9)	0.03 (0.2)	1.03 (7.4)	13.96 (100)
	**2022**	0	0.10 (0.9)	<0.01	0.84 (7.1)	2.51 (21.4)	0.26 (2.2)	4.36 (37.1)	0.14 (1.2)	1.84 (15.6)	0.08 (0.7)	1.62 (13.8)	11.74 (100)
**Guyana** ^a^	**2019**	0	1.17 (8.9)	0	6.08 (46.3)	0.16 (1.2)	1.82 (13.9)	2.37 (18.1)	0.02 (0.1)	1.25 (9.5)	<0.01	0.26 (2.0)	13.13 (100)
	**2020**	0	1.76 (6.7)	0	9.06 (34.8)	2.96 (11.4)	2.57 (9.9)	3.96 (15.2)	1.44 (5.5)	2.25 (8.6)	<0.01	2.03 (7.8)	26.02 (100)
	**2021**	0	0.57 (4.0)	0	5.07 (36.2)	0.15 (1.1)	1.69 (12.0)	3.95 (28.1)	0.01 (0.1)	2.47 (17.6)	0	0.11 (0.8)	14.02 (100)
	**2022**	0	0.11 (0.8)	0	6.05 (48.5)	0.3 (1.0)	1.50 (12.0)	2.19 (17.6)	0.01 (0.1)	2.13 (17.1)	<0.01	0.35 (2.8)	12.47 (100)
**Honduras**	**2019**	0	0.73 (5.6)	0	6.24 (47.8)	0.42 (3.3)	2.40 (18.4)	1.72 (13.2)	0.09 (0.7)	1.07 (8.2)	0.13 (1.0)	0.24 (1.9)	13.05 (100)
	**2020**	0	0.68 (5.6)	0	5.67 (46.5)	0.34 (2.8)	1.82 (15.0)	2.35 (19.3)	0.07 (0.6)	0.89 (7.3)	0.07 (0.6)	0.28 (2.3)	12.18 (100)
	**2021**	0	0.47 (5.2)	0	3.60 (39.4)	0.33 (3.7)	1.36 (15.0)	2.40 (26.3)	0.07 (0.7)	0.69 (7.6)	0.10 (1.1)	0.09 (1.0)	9.12 (100)
	**2022**	0	0.24 (3.4)	0	3.21 (45.1)	0.31 (4.3)	1.37 (19.3)	1.02 (14.3)	0.04 (0.5)	0.47 (6.7)	0.13 (1.8)	0.33 (4.6)	7.12 (100)
**Paraguay**	**2019** ^e^	0	<0.01	0	0.96 (28.5)	0.59 (17.6)	0.02 (0.7)	0.98 (28.9)	0.09 (2.6)	0.61 (18.0)	0.13 (3.8)	0	3.38 (100)
	**2020**	0	0.02 (0.3)	0	3.19 (37.7)	1.60 (19.0)	0.19 (2.3)	2.05 (24.3)	0.02 (0.2)	1.11 (13.2)	0.06 (0.7)	0.20 (2.3)	8.45 (100)
	**2021**	0	0.01 (0.1)	0	3.71 (38.3)	1.79 (18.4)	0.23 (2.4)	2.55 (26.3)	0.04 (0.4)	1.05 (10.8)	0.08 (0.8)	0.23 (2.4)	9.64 (100)
	**2022**	0	0.03 (0.2)	0	5.32 (45.4)	1.68 (14.3)	0.30 (2.6)	2.72 (23.2)	0.04 (0.3)	1.44 (12.3)	0.08 (0.7)	0.12 (1.1)	11.74 (100)
**Perú**	**2019**	0	0.98 (7.8)	0.03 (0.3)	4.49 (35.9)	1.31 (10.5)	0.76 (6.1)	2.00 (16.0)	0.27 (2.2)	1.75 (14.0)	0.41 (3.3)	0.49 (3.9)	12.50 (100)
	**2020**	<0.01	0.55 (6.8)	0.02 (0.3)	2.46 (30.5)	0.93 (11.5)	0.42 (5.3)	1.88 (23.3)	0.18 (2.3)	1.09 (13.5)	0.25 (3.2)	0.29 (3.5)	8.08 (100)
	**2021**	<0.01	0.69 (7.8)	0.02 (0.2)	2.64 (29.9)	1.19 (13.6)	0.52 (5.9)	1.75 (19.8)	0.22 (2.5)	1.14 (12.9)	0.31 (3.5)	0.35 (3.9)	8.81 (100)
	**2022**	0	0.78 (7.6)	0.01 (0.1)	3.54 (34.2)	1.26 (12.2)	0.56 (5.4)	2.01 (19.5)	0.21 (2.0)	1.28 (12.4)	0.31 (3.0)	0.37 (3.6)	10.33 (100)
**Saint Kitts**	**2022**	0.02 (0.8)	0.16 (6.1)	0	0.48 (18.9)	0.60 (23.5)	0.18 (7.2)	0.25 (9.7)	0.03 (1.0)	0.55 (21.5)	0.11 (4.2)	0.18 (7.0)	2.55 (100)
**Nevis** ^b^	**2021**	0	0.22 (6.3)	0	0.95 (26.9)	0.65 (18.2)	0.35 (9.7)	0.62 (17.5)	0.12 (3.4)	0.29 (8.2)	0.07 (1.9)	0.28 (7.9)	3.55 (100)
**Trinidad and Tobago** ^a,b,c^	**2019**	NR	NR	NR	2.38 (38.4)	1.84 (29.7)	0.30 (4.8)	0.39 (6.3)	NR	1.27 (20.5)	NR	NR	6.19 (100)
	**2020**	NR	NR	NR	3.12 (45.9)	1.42 (20.9)	1.26 (18.5)	0.27 (4.0)	NR	0.72 (10.6)	NR	NR	6.80 (100)
	**2021**	NR	NR	NR	2.02 (43.8)	1.47 (31.9)	0.27 (5.9)	0.33 (7.2)	NR	0.52 (11.3)	NR	NR	4.61 (100)

NR: No reported data. ^a^ Guyana AMC data was limited to Region 2, and for Trinidad and Tobago to the Eastern Regional Health Authority (ERHA). ^b^ The data presented corresponds to the total consumption (hospital + community). ^c^ Only monitored a specific set of antimicrobials, chosen for convenience. ^d^ There is a potential underestimation of AMC for Costa Rica during the year 2022, as the informatic systems of the CCSS were impacted by a cyberattack during that year. ^e^ The 2019 AMC data was limited to hospital level.

**Table 4:** AMC categorized according to WHO AWaRe classification, for each country and year, expressed in DID (DDD/1000 inhab./day) and as a percentage (%) of the total consumption.

		Access	Watch	Reserve	Without Classification	Total
**Argentina**	**2019**	28.33 (78.1)	7.72 (21.3)	0.03 (0.1)	0.19 (0.5)	36.26 (100)
	**2020**	11.36 (65.2)	5.95 (34.2)	0.03 (0.1)	0.08 (0.4)	17.42 (100)
	**2021**	10.81 (63.2)	6.18 (36.1)	0.04 (0.2)	0.09 (0.5)	17.12 (100)
	**2022**	15.71 (63.5)	8.46 (34.2)	0.03 (0.1)	0.54 (2.2)	24.74 (100)
**Barbados**	**2020**	11.18 (72.6)	4.21 (27.3)	0	0	15.40 (100)
	**2022**	15.30 (84.0)	2.83 (15.6)	0.07 (0.4)	0	18.20 (100)
**Brazil**	**2019**	4.11 (58.3)	2.94 (41.7)	<0.01	0	7.05 (100)
	**2020**	3.51 (49.8)	3.55 (50.2)	<0.01	0	7.06 (100)
**Chile**	**2019**	4.00 (62.3)	2.40 (37.5)	0.01 (0.1)	0.01 (0.1)	6.41 (100)
	**2020**	3.51 (62.0)	2.15 (37.8)	0.01 (0.2)	<0.01	5.67 (100)
	**2021**	2.65 (64.3)	1.45 (35.3)	0.01 (0.2)	<0.01 (0.1)	4.12 (100)
	**2022**	3.20 (59.8)	2.13 (39.8)	0.02 (0.3)	<0.01	5.34 (100)
**Colombia**	**2019**	14.81 (74.4)	4.97 (25.0)	0.03 (0.2)	0.09 (0.4)	19.90 (100)
	**2020**	15.22 (67.6)	7.06 (31.4)	0.03 (0.1)	0.20 (0.9)	22.51 (100)
	**2021**	17.32 (65.7)	8.76 (33.2)	0.04 (0.2)	0.23 (0.9)	26.35 (100)
	**2022**	12.22 (65.9)	5.99 (32.3)	0.04 (0.2)	0.29 (1.6)	18.54 (100)
**Costa Rica**	**2019**	10.59 (83.1)	2.16 (17.0)	0.01 (0.1)	0	12.76 (100)
	**2020**	7.62 (85.8)	1.25 (14.1)	0.01 (0.1)	0	8.89 (100)
	**2021**	7.93 (86.5)	1.24 (13.5)	0.01 (0.1)	0	9.17 (100)
	**2022** ^d^	7.31 (86.0)	1.18 (13.9)	0.01 (0.1)	0	8.50 (100)
**Cuba**	**2021**	7.31 (52.4)	6.61 (47.3)	<0.01	0.04 (0.3)	13.96 (100)
	**2022**	4.77 (40.7)	6.96 (59.3)	<0.01	<0.01	11.74 (100)
**Guyana** ^a^	**2019**	9.49 (72.3)	3.64 (27.7)	0	0	13.13 (100)
	**2020**	17.29 (66.4)	8.73 (33.5)	0	0	26.02 (100)
	**2021**	7.67 (54.7)	6.35 (45.3)	0	0	14.02 (100)
	**2022**	8.20 (65.6)	4.27 (34.2)	0	0	12.47 (100)
**Honduras**	**2019**	10.07 (77.2)	2.98 (22.8)	<0.01	0	13.05 (100)
	**2020**	8.79 (72.1)	3.39 (27.9)	<0.01	0	12.18 (100)
	**2021**	5.83 (63.9)	3.29 (36.0)	<0.01	0	9.12 (100)
	**2022**	5.44 (76.4)	1.68 (23.6)	<0.01	0	7.12 (100)
**Paraguay**	**2019** ^e^	0.66 (19.5)	2.02 (59.8)	0.03 (0.8)	0.68 (20.0)	3.38 (100)
	**2020**	2.52 (29.9)	3.85 (45.6)	<0.01	2.08 (24.6)	8.45 (100)
	**2021**	2.83 (29.2)	4.51 (46.5)	0.01 (0.1)	2.35 (24.2)	9.64 (100)
	**2022**	3.57 (30.4)	4.81 (41.0)	0.01 (0.1)	3.35 (28.5)	11.74 (100)
**Perú**	**2019**	8.33 (66.6)	4.16 (33.3)	0.01 (0.1)	0	12.50 (100)
	**2020**	4.71 (58.3)	3.36 (41.6)	0.01 (0.1)	<0.01	8.08 (100)
	**2021**	5.37 (61.0)	3.42 (38.9)	0.02 (0.2)	<0.01	8.81(100)
	**2022**	6.53 (63.2)	3.78 (36.6)	0.02 (0.2)	0	10.33 (100)
**Saint Kitts**	**2022**	1.23 (48.2)	1.30 (51.1)	0	0.02 (0.8)	2.55 (100)
**Nevis** ^b^	**2021**	2.09 (58.9)	1.46 (41.1)	0	0	3.55 (100)
**Trinidad and Tobago** ^a,b,c^	**2019**	2.69 (43.5)	3.50 (56.5)	0	0	6.19 (100)
	**2020**	4.39 (64.6)	2.41 (35.3)	0	0	6.80 (100)
	**2021**	2.29 (49.7)	2.32 (50.3)	0	0	4.61 (100)

^a^ Guyana AMC data was limited to Region 2, and for Trinidad and Tobago to the Eastern Regional Health Authority (ERHA). ^b^ The data presented corresponds to the total consumption (hospital + community). ^c^ Only monitored a specific set of antimicrobials, chosen for convenience. ^d^ There is a potential underestimation of AMC for Costa Rica during the year 2022, as the informatic systems of the CCSS were impacted by a cyberattack during that year. ^e^ The 2019 AMC data was limited to hospital level.

## Data Availability

All data will be open and free available through the UNLP SEDECI-Repository https://sedici.unlp.edu.ar/.
